# Statistical Assessment of Solvent Mixture Models Used for Separation of Biological Active Compounds

**DOI:** 10.3390/molecules13081617

**Published:** 2008-08-11

**Authors:** Sorana D. Bolboacă, Elena M. Pică, Claudia V. Cimpoiu, Lorentz Jäntschi

**Affiliations:** 1 “Iuliu Haţieganu” University of Medicine and Pharmacy Cluj-Napoca, Department of Medical Informatics and Biostatistics, 6 Louis Pasteur, 400349 Cluj-Napoca, Romania; 2 Technical University of Cluj-Napoca, 103-105 Muncii Bvd, 400641 Cluj-Napoca, Romania; E-mails: pica@chimie.utcluj.ro, lori@chimie.utcluj.ro; 3 “Babeş-Bolyai”University, Department of Analytical Chemistry, 11 Arany Janos, 400028 Cluj-Napoca, Romania; E-mail: ccimpoiu@chem.ubbcluj.ro

**Keywords:** Mathematical model, chromatographic response functions, statistical assessment, correlation

## Abstract

Two mathematical models with seven and six parameters have been created for use as methods for identification of the optimum mobile phase in chromatographic separations. A series of chromatographic response functions were proposed and implemented in order to assess and validate the models. The assessment was performed on a set of androstane isomers. Pearson, Spearman, Kendall tau-a,b,c and Goodman-Kruskal correlation coefficients were used in order to identify and to quantify the link and its nature (quantitative, categorical, semi-quantitative, both quantitative and categorical) between experimental values and the values estimated by the mathematical models. The study revealed that the six parameter model is valid and reliable for five chromatographic response factors (retardation factor, retardation factor ordered ascending by the chromatographic peak, resolution of pairs of compound, resolution matrix of successive chromatographic peaks, and quality factor). Furthermore, the model could be used as an instrument in analysis of the quality of experimental data. The results obtained by applying the model with six parameters for deviations of rank sums suggest that the data of the experiment no. 8 are questionable.

## Introduction

Chromatographic analysis, defined as the technique used for separation of a mixture of compounds by their distribution between two phases, was invented in 1901 by the Russian botanist Mikhail Semyonovich Tsvet, during his research on plant pigments [1]. He used liquid-adsorption column chromatography with calcium carbonate as adsorbent and petroleum ether/ethanol mixtures as eluent to separate chlorophylls and carotenoids. The method was presented at the XI Congress of Naturalists and Doctors in St. Petersburg in 1901 but the term “chromatography” was used for the first time in 1906 in a paper published in the Berichte der Deutschen Botanischen Gesellschaft Journal [2].

Today, chromatography is a separation method frequently used in chemistry [3,4], biology [5,6], and medicine [7,8] as an analytical technique. The choice of the optimum mobile phase composition is the most difficult and most time-consuming task [9,10]. Optimization procedures have been developed by many researchers in order to obtain the optimum mobile phase. Some geometrical or mathematical models were proposed and assessed [11,12,13,14,15]. Moreover, some optimization methods that use neural networks have been introduced [16,17].

A series of experiments were performed and the optimum mobile phases were obtained for different classes of compounds (steroids [18,19,20] and benzodiazepines [13,17]). The aim of the present research was to assess through statistical parameters and tests two mathematical models created for optimization of mobile phase in chromatographic separation applied on a set of androstane isomers.

## Material and Methods

### Experimental Measurements

A set of five previously investigated androstane isomers (5α-androstane-3β-ol, 5α-androstane-3α-ol, 5α-androstane-17β-ol, 5β-androstane-3α,17β-diol, and 5β-androstane-3β,17β-diol) [20] was included into the study. Eleven experimental values were considered (see Table 1). The 11^th^ experiment was a result of an optimization method applied on an objective function that suggests the composition of the optimum mobile phase as 55:19:26 (trichloromethane:propanone:petroleum ether).

The mathematical models presented in Eqs. (1.1) and (1.2) were used in some studies, including in the investigation of the set of androstane isomers presented in [20,21]:

M7(x_1_,x_2_,x_3_) = a_1_x_1_ + a_2_x_2_ + a_3_x_3_ + a_4_x_1_x_2_ + a_5_x_1_x_3_ + a_6_x_2_x_3_ + a_7_x_1_x_2_x_3_ (**1.1**)

M6(x_1_,x_2_,x_3_) = a_1_x_1_ + a_2_x_2_ + a_3_x_3_ + a_4_x_1_x_2_ + a_5_x_1_x_3_ + a_6_x_2_x_3_ (**1.2**)

where M7, M6 = estimator and predictor of the selected chromatographic parameter; x_1_, x_2_, and x_3_ = molar fractions of the three solvents (where x_1_ + x_2_ + x_3_ = 1); a_1_, a_2_, a_3_, a_4_, a_5_, a_6_, and a_7_ = model coefficients; first determined based on the best estimation of the selected chromatographic parameter (using 7 experiments for M7; 6 experiments for M6) and then used to predict used chromatographic parameter for any composition of the mobile phase (used for not included in model experiments).

The concentration of the solvents into the mixture and the experimental data are presented in Table 1. The chromatography for experiments 1 - 11 was performed on 5×10 cm glass HPTLC plates pre-coated with silica gel 60 F_254_ (Merck) (Table 1).

**Table 1:** Experimental data: androstane isomers.

No.	TCM:Prop:PE^*^	L	l_1_	w_1_	l_2_	w_2_	l_3_	w_3_	l_4_	w_4_	l_5_	w_5_
1	33:33:33	8.70	6.65	0.48	7.36	0.35	7.26	0.23	4.00	0.38	4.76	0.98
2	0:0:100	8.83	0.00	0.42	0.00	0.44	0.00	0.22	0.00	0.25	0.00	0.21
3	0:100:0	8.75	8.29	0.37	8.49	0.26	8.49	0.11	7.93	0.28	7.79	0.59
4	100:0:0	9.00	1.21	0.62	2.05	0.45	1.43	0.41	0.05	0.23	0.19	0.30
5	50:0:50	8.93	0.54	0.56	0.98	0.38	0.68	0.27	0.00	0.26	0.00	0.25
6	50:50:0	8.84	6.71	0.55	7.12	0.31	7.05	0.20	5.31	0.36	5.56	0.69
7	0:50:50	8.76	8.44	0.36	8.56	0.11	8.56	0.05	7.35	0.31	7.20	1.38
8	10:10:80	8.86	3.49	0.60	4.71	0.42	4.51	0.28	0.53	0.27	0.64	1.41
9	80:10:10	8.87	5.08	0.69	6.71	0.51	6.06	0.34	1.01	0.32	2.32	0.63
10	10:80:10	8.82	8.24	0.52	8.41	0.24	8.46	0.14	7.38	0.32	7.27	0.96
11	55:19:26	18.95	3.43	0.82	5.86	1.16	11.52	1.43	13.44	1.25	14.38	1.32
^*^ Trichloromethane (CHCl_3_ [mL]) : Propanone (CH_3_COCH_3_ [mL]) : Petroleum ether ([mL]) L = migration distance of the eluent *e* l_i_ = migration distance of *i^the^* compound in eluent *e* (where *i = 1, 2, …5*) w_i_ = spot width of *i^th^* compound (where *i = 1, 2, …, 5*)												

### Statistical Validation

The statistical hypothesis of the research was as follows: the mathematical model with seven parameters proposed by Eq (1.1.), and the model with six parameters proposed by Eq (1.2) are reliable and valid models for estimation of a given response function (H_0_).

A series of parameters were defined and proposed to be used as estimators of the separation quality (see Table 2). The terms and symbols used in the assessment of the mathematical model are as were established by the International Union of Pure and Applied Chemistry (IUPAC) in the Nomenclature for Chromatography [22].

The model is not considered reliable and valid if the null hypothesis (H_0_) is rejected at a significance level of 5% in the investigation of the response factors (see Table 2). Pearson (r), Spearman (ρ), Kendall (τ-a,b,c) and Gamma (Γ) correlation coefficients [23,24,25] were used in order to identify and to quantify the nature of the link (quantitative, categorical, semi-quantitative, quantitative and categorical) between experimental and estimated values. The correlation approach was choose for analysis of the quality of the models (Eq (1.1), and Eq (1.2), respectively) due to its ability of identification of linear relationship between two variable (in our case the experimental and estimated values by the proposed mathematical models from Eq (1.1) and Eq (1.2), respectively).

**Table 2:** Chromatographic response function for statistical assessment.

Parameter	Formula	Eq.	Notes	
Retardation factors (RF) matrix	RF(i,e) = l(i,e)/l(e)	(2)	i	a separated compound
			e	the mobile phase
			l(i,e)	migration distance of i in e
			l(e)	migration distance of e
Ordered RF	RFO(i,e) = 2·(l_o_(i+1,e)-l_o_(i,e))/l(e)	(3)	l_o_(i,e)	i^th^ migration coordinate in the list of migration, ordered by length
Resolution matrix	RSM(i,j,e) = 2·(l(i,e)-l(j,e))/(w(i,e)+w(j,e))	(4)	j	a separated compound
			w(i,e)	spot width of i
			w(j,e)	spot width of j
Resolution of adjacent spots matrix	RSO(i,e) = 2·(l_o_(i+1,e)-l_o_(i,e))/(w(i+1,e)+w(i,e))	(5)	l_o_(i,e)	i^th^ migration coordinate in the list of migration, ordered by length
Number of components	nc(e) = Σ_i_ 1 | l_o_(i+1,e)-l_o_(i,e)>(w(i+1,e)+w(i,e))/8	(6)	nc(e)	number of components observed in e at least 1σ (σ = standard deviation)
Maximum number of components	mnc = max_e_ nc(e)	(7)	mnc	from all experimented mobile phases (or previous knowledge)
Retardation factors deviation	RFD(e) = √(∑_i_ (ΔRF(i,e)-1/mnc)^2^/√nc(e)(nc(e)+1)	(8)	1/mnc	theoretical difference between two retardation factors
			ΔRF(i,e)	RFO(i+1,e)-RFO(i,e)
Informational energy	IEne(e) = mnc^2^ - Σ_i _(n_i_)^2^	(9)	n_i_	number of compounds that migrate into i^th^ equidistant interval from mnc intervals
Informational entropy	IEnt(e) = Σ_i _(n_i_)log_2_(n_i_)	(10)		
Resolution sum	RSS(e) = ∑_i_ RSO(i,e)	(11)	RSS(e)	average indicator for separation
Effective plates number squared root	QN_eff_(e) = 4·l(e)/(Σ_i_ w(e,i))	(12)	QN_eff_(e)	average indicator for a hypothetic quantitative analysis
Resolution divided by the number of effective plates	RSP(e) = 25·RSS(e)/QN_eff_(e)	(13)	RSP(e)	composite indicator for separation expressed as proportion; note that 4·RSS(e) → QN_eff_(e) for an ideal separation
Average resolution for separation	RSA(e) = RSS(e)/nc(e)	(14)	RSA(e)	average indicator for separation
Relative resolution product	RRP(e) = Π_i_ RSO(i,e)/ Σ_i_ RSO(i,e)	(15)	RRP(e)	average indicator for separation
Minkowski type mean of resolutions	RSR(e) = (∑_i_ (RSO(i,e))^1/p^/nc(e))^p^; p = 2	(16)	RSR(e)	is better descriptor for separation than RSA
Quality factor	QF(e) = min_i,j_ RSM(i,j,e) = min_i_ RSO(i,e)	(17)	QF(e)	worst one define the resolution of separation
Notes: ÷ Informational energy is a quality factor computed by the Logit method, which is equal with 0 for an ideal separation ÷ Informational energy is a quality factor which is equal with 0 for an ideal separation ÷ Part of the entries in Table 2 were previously used in the literature, with different names; thus, relating [20], RFD was reported as Sm.				

A significant correlation coefficient (at a significance level of 5%) sustains the validity of the model from Eq (1.1) and Eq (1.2) in the estimation of chromatographic response factors of interest. An online resource hosted by AcademicDirect was used in order to calculate correlation coefficients and associated statistic parameters (http://l.academicdirect.org/Statistics/linear_dependence/).

The difference between two correlation coefficients was tested by using Statistica 6.0 software, Basic Statistics and Tables - Differences tests - Differences between two correlation coefficients.

If Pearson’s correlation coefficient is much smaller than Spearman’s correlation coefficient applied to the same variables, it can be concluded that the variable of interest correlated consistently (if both are statistically significant), but not in a linear quantitative manner (i.e. may not be linear; may not be quantitative).

A linear relationship between experimental and estimated values (given by Eq (1.1) and Eq (1.2), respectively) was considered to be proven when Pearson’s and Spearman’s correlation coefficients had similar values (i.e. there is no significant statistical difference) that are statistically significant different from zero. The Kendall’s as well as Gamma correlation coefficients make no assumption whatsoever about the distribution of the investigated values (it is a ratio that investigate the “concordant” and “discordant” pairs). Opposite to Pearson's correlation coefficient, Kendall’s tau correlation coefficients are measures of correlation between two ordinal-level variables. When Kendall’s tau correlation coefficient are applied, for more concordant than discordant pairs the value of the coefficient is positive, for equal concordant and discordant pairs the coefficient is zero.

## Results and Discussion

A series of results were obtained by applying the parameters described in Table 2 on the set of androstane isomers. Four response functions were computed as the measurement space (Eq (2)-Eq (5)): retardation factor, retardation factors ordered ascending by the chromatographic peak, resolution of separation, and resolution of separation of successive peaks. The proposed response functions (Eq (6)-Eq (17)) for investigation of the reliability and validity of the model presented in Eq (1.1) and Eq (1.2) where computed based on the results of Eq (2)-Eq (5).

The retardation factor matrix (the response function of the compound separation in chromatography, defined as the relative migration distance) for each investigated compound is presented in Table 3.

**Table 3:** Matrix of retardation factor: experimental vs estimated.

No.	Experimental					Estimated by Eq(1.1)					Estimated by Eq(1.2)				
	AI1	AI2	AI3	AI4	AI5	AI1	AI2	AI3	AI4	AI5	AI1	AI2	AI3	AI4	AI5
1	0.764	0.845	0.834	0.460	0.547						0.006	0.021	0.134	0.159	0.228
2	0.000	0.000	0.000	0.000	0.000										
3	0.947	0.970	0.970	0.906	0.890										
4	0.134	0.228	0.159	0.006	0.021										
5	0.060	0.110	0.076	0.000	0.000										
6	0.759	0.805	0.798	0.601	0.629										
7	0.963	0.977	0.977	0.839	0.822										
8	0.394	0.532	0.509	0.060	0.072	0.291	0.314	0.308	0.203	0.216	0.215	0.219	0.271	0.279	0.284
9	0.573	0.756	0.683	0.114	0.262	0.309	0.393	0.347	0.139	0.174	0.157	0.172	0.289	0.317	0.364
10	0.934	0.954	0.959	0.837	0.824	1.017	1.052	1.053	0.878	0.891	0.882	0.903	0.997	1.024	1.022
11	0.181	0.309	0.608	0.709	0.759	0.505	0.591	0.565	0.253	0.324	0.309	0.323	0.438	0.465	0.491
AI1 = 5α-androstane-3β-ol; AI2 = 5α-androstane-3α-ol AI3 = 5a-androstane-17β-ol; AI4 = 5β-androstane-3α,17β-diol; AI5 = 5β-androstane-3β,17β-diol															

The experimental and estimated retardation factor had identical values for first experiments because these were used to construct the model: the coefficients of the model with seven parameters proposed by Eq (1.1) were calculated based on experiments 1...7 presented in Table 1. A similar procedure was followed for experiments 2...7 included in the model with six parameters proposed by Eq (1.2). The differences between experimental and estimated values (for the experiments not included in learning set) showed that the great variation was obtained by the experiment no. 11. The difference varied from -0.324 (AI1 = 5α-androstane-3β-ol, experiment no. 11, model M7) to 0.584 (AI4 = 5β-androstane-3α,17β-diol, experiment no. 11, model M6). The estimated values were greater than the experimental values in half of the cases. Systematically, the estimated values were greater than experimental values for experiment no. 10 - Eq (1.1) and lower than experimental values for experiment no. 1 - Eq (1.2).

The correlation analysis on experimental versus estimated retardation factor give the results presented in Table 4. The analysis of the values of correlation coefficients (all values are higher than 0.5 and all correlation are statistically significant p < 1.39·10^-2^) revealed that the models from Eq (1.1) and Eq (1.2) have good abilities in estimation of chromatographic retardation factor.

**Table 4:** Correlation analysis on retardation factor: experimental versus estimated.

Name	Correlation coefficient	p-value	Statistical parameter
**Eq(1.1), n = 20**			
Pearson	r = 0.7214	3.31·10^-4^	t_Prs,1_ = 4.42
Spearman	ρ = 0.7789	5.19·10^-5^	t_Spm,1_ = 5.27
Semi-Q	r_sQ_ = 0.7496	1.42·10^-4^	t_sQ_ = 4.80
Kendall τa	τ_Ken,a_ = 0.6316	9.89·10^-5^	Z_Ken,τa_ = 3.89
Kendall τb	τ_Ken,b_ = 0.6316	9.89·10^-5^	Z_Ken,τb_ = 3.89
Kendall τc	τ_Ken,c_ = 0.6000	2.17·10^-4^	Z_Ken,τc_ = 3.70
Gamma	Γ = 0.6316	1.39·10^-2^	Z_Γ_ = 2.46
**Eq (1.2), n = 25**			
Pearson	r = 0.8292	3.02·10^-7^	t_Prs,1_ = 7.11
Spearman	ρ = 0.9008	8.45·10^-10^	t_Spm,1_ = 9.95
Semi-Q	r_sQ_ = 0.8642	2.58·10^-8^	t_sQ_ = 8.24
Kendall τa	τ_Ken,a_ = 0.7667	7.80·10^-8^	Z_Ken,τa_ = 5.37
Kendall τb	τ_Ken,b_ = 0.7667	7.80·10^-8^	Z_Ken,τb_ = 5.37
Kendall τc	τ_Ken,c_ = 0.7360	2.51·10^-7^	Z_Ken,τc_ = 5.16
Gamma	Γ = 0.7667	3.82·10^-5^	Z_Γ_ = 4.12

The highest correlation coefficient is obtained by the Spearman method and leads to the idea that the retardation factor is a categorical not a quantitative variable. Statistically, there was not identified any significant difference between correlation coefficients obtained by different methods, neither for Eq (1.1) nor for Eq (1.2) (the lowest value of 0.3465 was obtained in comparison of Spearman and Kendall - Eq (1.1); a p-value of 0.0780 was obtained in comparison of Spearman and Kendall τc - Eq (1.2)).

The retardation matrix ordered ascending for each chromatographic peak was obtained based on Eq (3). The results for experimental and estimated determinations are presented in Table 5. The correspondence between each peak and the compound was known on the data presented in Table 5. The difference between experimental and estimated values varied from -0.155 (1^st^ peak, experiment no. 8, Eq (1.2)) to 0.363 (5^th^ peak, experiment no. 9, Eq (1.1)) and 0.675 (4^th^ peak, experiment no. 1, Eq (1.2)). Systematically, the estimated values were higher than experimental values for experiment no. 10, for both Eq (1.1) and Eq (1.2). The correlation analysis between experimental and estimated values (by Eq (1.1) and Eq (1.2), respectively) leads to the results presented in Table 6. 

**Table 5:** Matrix of retardation factors ordered by the chromatographic peak: experimental vs estimated.

No.	Experimental peak					Estimated peak by Eq(1.1)					Estimated peak by Eq(1.2)				
	1^st^	2^nd^	3^rd^	4^th^	5^th^	1^st^	2^nd^	3^rd^	4^th^	5^th^	1^st^	2^nd^	3^rd^	4^th^	5^th^
1	0.460	0.547	0.764	0.834	0.845						0.006	0.021	0.134	0.159	0.228
2	0.000	0.000	0.000	0.000	0.000										
3	0.890	0.906	0.947	0.970	0.970										
4	0.006	0.021	0.134	0.159	0.228										
5	0.000	0.000	0.060	0.076	0.110										
6	0.601	0.629	0.759	0.798	0.805										
7	0.822	0.839	0.963	0.977	0.977										
8	0.060	0.072	0.394	0.509	0.532	0.200	0.219	0.291	0.308	0.314	0.215	0.219	0.271	0.279	0.284
9	0.114	0.262	0.573	0.683	0.756	0.141	0.172	0.309	0.347	0.393	0.157	0.172	0.289	0.317	0.364
10	0.824	0.837	0.934	0.954	0.959	0.866	0.902	1.017	1.053	1.052	0.882	0.903	0.997	1.024	1.022
11	0.181	0.309	0.608	0.709	0.759	0.256	0.321	0.505	0.565	0.591	0.309	0.323	0.438	0.465	0.491
L = migration distance of the eluent															

The difference between two correlation coefficients was tested and the results are presented in Table 7. By analyzing the results from Table 7 it can be seen that the investigated chromatographic response function is more like to be a categorical variable but not a rank variable then a quantitative variable (the Spearman rank correlation coefficient is statistically significant greater that Kendall τc applied on Eq (1.1)). The data presented in Table 6 revealed that all correlation coefficient were statistically significant (p ≤ 3.82·10^-5^). Thus, it can be concluded that the link between experimental and estimated by Eq (1.1) and Eq (1.2) data are linear related and sustain the validity of the models from Eq (1.1) and Eq (1.2) for this chromatographic response function.

**Table 6:** Correlation analysis on retardation factor ordered ascending by the chromatographic peak (experimental vs estimated values).

Name	Correlation coefficient	p-value	Statistical parameter
**Eq(1.1), n = 20**			
Pearson	r = 0.8654	8.38·10^-7^	t_Prs,1_ = 7.33
Spearman	ρ = 0.9579	3.39·10^-11^	t_Spm,1_ = 14.15
Semi-Q	r_sQ_ = 0.9105	2.53·10^-8^	t_sQ_ = 9.34
Kendall τa	τ_Ken,a_ = 0.8526	1.47·10^-7^	Z_Ken,τa_ = 5.26
Kendall τb	τ_Ken,b_ = 0.8526	1.47·10^-7^	Z_Ken,τb_ = 5.26
Kendall τc	τ_Ken,c_ = 0.8100	5.94·10^-7^	Z_Ken,τc_ = 4.99
Gamma	Γ = 0.8526	7.42·10^-6^	Z_Γ_ = 4.48
**Eq(1.2), n = 25**			
Pearson	r = 0.8292	3.02·10^-7^	t_Prs,1_ = 7.11
Spearman	ρ = 0.9008	8.45·10^-10^	t_Spm,1_ = 9.95
Semi-Q	r_sQ_ = 0.8642	2.58·10^-8^	t_sQ_ = 8.24
Kendall τa	τ_Ken,a_ = 0.7667	7.80·10^-8^	Z_Ken,τa_ = 5.37
Kendall τb	τ_Ken,b_ = 0.7667	7.80·10^-8^	Z_Ken,τb_ = 5.37
Kendall τc	τ_Ken,c_ = 0.7360	2.51·10^-7^	Z_Ken,τc_ = 5.16
Gamma	Γ = 0.7667	3.82·10^-5^	Z_Γ_ = 4.12

**Table 7:** Matrix of p-values: test of difference between two correlation coefficients.

	Pearson	Spearman	Semi-Q	Kendall τa	Kendall τb	Kendall τc	Gamma
Eq(1.1), n = 20		Eq(1.2), n = 25					
**Pearson**	1.0000	0.3510	0.7287	0.5805	0.5805	0.4346	0.5805
**Spearman**	0.0824	1.0000	0.5559	0.1408	0.1408	0.0903	0.1408
**Semi-Q**	0.5743	0.2305	1.0000	0.3699	0.3699	0.2614	0.3699
**Kendall τa**	0.7416	0.0519	0.2468	1.0000	0.3699	0.2614	0.3699
**Kendall τb**	0.7416	0.0519	0.2468	1.0000	1.0000	1.0000	0.8178
**Kendall τc**	0.3783	0.0223	0.0890	0.5803	0.5803	1.0000	0.8178
**Gamma**	0.7416	0.0519	0.2468	1.0000	1.0000	0.5803	1.0000

The resolution of separation between any two investigated androstane isomers could be considered one of the top-three quality measurements of a chromatographic separation. The resolution matrix between pairs of androstane isomers are presented in Table 8. The highest the resolution value between two compounds, the better the separation is considered.

The difference between experimental and estimated values for experiments 8-11 varied from to -7.232 (the resolution between 5β-androstane-3α,17β-diol and 5β-androstane-3β,17β-diol, experiment no. 8, Eq (1.1)) to 11.827 (the resolution between 5a-androstane-17β-ol and 5β-androstane-3α,17β-dio, experiment no.8, Eq (1.2)). With a single exception, the estimated exceed the experimental values (experiment no. 10, for both Eq (1.1) and Eq (1.2), respectively). The results of the correlation analysis are presented in Table 9.

**Table 8:** Resolution matrix of pairs of compounds: experimental vs estimated.

No.	AI1-AI2	AI1-AI3	AI1-AI4	AI1-AI5	AI2-AI3	AI2-AI4	AI2-AI5	AI3-AI4	AI3-AI5	AI4-AI5
**Experimental**										
1	1.687	1.718	6.163	2.589	0.310	9.178	3.895	10.689	4.132	1.118
2	0.000	0.000	0.000	0.000	0.000	0.000	0.000	0.000	0.000	0.000
3	0.635	0.833	1.108	1.042	0.000	2.074	1.647	2.872	2.000	0.322
4	1.570	0.427	2.729	2.217	1.442	5.882	4.960	4.313	3.493	0.528
5	0.936	0.337	1.317	1.333	0.923	3.063	3.111	2.566	2.615	0.000
6	0.953	0.907	3.077	1.855	0.275	5.403	3.120	6.214	3.348	0.476
7	0.511	0.585	3.254	1.425	0.000	5.762	1.826	6.722	1.902	0.178
8	2.392	2.318	6.805	2.836	0.571	12.116	4.448	14.473	4.580	0.131
9	2.717	1.903	8.059	4.182	1.529	13.735	7.702	15.303	7.711	2.758
10	0.447	0.667	2.048	1.311	0.263	3.679	1.900	4.696	2.164	0.172
11	2.455	7.191	9.671	10.234	4.371	6.290	6.871	1.433	2.080	0.732
Estimated by Eq(1.1)										
8	0.512	0.456	1.968	0.891	0.178	3.211	1.402	3.639	1.409	0.207
9	1.515	0.786	3.447	2.253	1.062	6.405	4.542	5.912	3.770	0.586
10	0.872	1.082	3.081	1.620	-0.004	5.028	2.327	6.269	2.734	0.521
11	1.681	1.380	5.042	2.480	0.677	7.971	4.292	8.665	4.142	0.901
Estimated by Eq(1.2)										
1	0.822	0.673	2.973	1.688	0.372	5.440	2.847	6.091	2.885	0.196
8	0.325	0.231	1.279	0.696	0.191	2.404	1.176	2.646	1.14	0.008
9	1.328	0.56	2.758	2.058	1.076	5.597	4.316	4.919	3.501	0.387
10	0.685	0.857	2.392	1.426	0.01	4.22	2.101	5.276	2.465	0.322
11	1.046	0.613	2.702	1.819	0.722	5.228	3.523	5.292	3.228	0.225
AI1 = 5α-androstane-3β-ol AI2 = 5α-androstane-3α-ol AI3 = 5a-androstane-17β-ol AI4 = 5β-androstane-3α,17β-diol AI5 = 5β-androstane-3β,17β-diol										

Two out of seven correlation coefficients were not statistically significant according to the Pearson and Gamma correlation coefficients. The values of the other correlation coefficients were not significantly different by each other (the lowest p-value was obtained when Spearman and Kendall τc were compared with values of 0.2578 - Eq (1.1), and 0.1862 - Eq (1.2), respectively).

The experimental matrices of the successive chromatographic peaks and those estimated by the models Eq (1.1) and Eq (1.2) are presented in Table 10. The difference between the experimental and estimated values varied from -0.635 (1^st^ peak - experiment no. 9, Eq (1.1)) and 2.172 (2^nd^ peak - experiment no. 10, Eq (1.1)). In most cases (four out of five), the estimated by Eq (1.1) values were greater than the experimental values for experiment no. 10.

**Table 9:** Correlation analysis on resolutions: experimental vs estimated.

Name	Correlation coefficient	p-value	Statistical parameter
**Estimated by Eq(1.1), n = 40**			
Pearson	r = 0.5173	6.30·10^-4^	t_Prs,1_ = 3.72
Spearman	ρ = 0.6214	1.88·10^-5^	t_Spm,1_ = 4.89
Semi-Q	r_sQ_ = 0.5670	1.36·10^-4^	t_sQ_ = 4.24
Kendall τa	τ_Ken,a_ = 0.4462	5.02·10^-5^	Z_Ken,τa_ = 4.05
Kendall τb	τ_Ken,b_ = 0.4462	5.02·10^-5^	Z_Ken,τb_ = 4.05
Kendall τc	τ_Ken,c_ = 0.4350	7.71·10^-5^	Z_Ken,τc_ = 3.95
Gamma	Γ = 0.4462	7.05·10^-2^	Z_Γ_ = 1.81
**Estimated by Eq(1.2), n = 50**			
Pearson	r = 0.6185	1.70·10^-6^	t_Prs,1_ = 5.45
Spearman	ρ = 0.6786	6.12·10^-8^	t_Spm,1_ = 6.40
Semi-Q	r_sQ_ = 0.6478	3.67·10^-7^	t_sQ_ = 5.89
Kendall τa	τ_Ken,a_ = 0.4939	4.18·10^-7^	Z_Ken,τa_ = 5.06
Kendall τb	τ_Ken,b_ = 0.4939	4.18·10^-7^	Z_Ken,τb_ = 5.06
Kendall τc	τ_Ken,c_ = 0.4840	7.07·10^-7^	Z_Ken,τc_ = 4.96
Gamma	Γ = 0.4939	1.24·10^-2^	Z_Γ_ = 2.50

**Table 10:** Resolution matrices of successive chromatographic peaks: experimental vs estimated.

No.	Experimental peak					Estimated peak by Eq(1.1)					Estimated peak by Eq(1.2)				
	1^st^	2^nd^	3^rd^	4^th^	5^th^	1^st^	2^nd^	3^rd^	4^th^	5^th^	1^st^	2^nd^	3^rd^	4^th^	5^th^
1	1.118	2.589	1.718	0.310	0.845						0.196	2.487	0.673	0.372	0.196
2	0.000	0.000	0.000	0.000	0.000										
3	0.322	1.108	0.833	0.000	0.970										
4	0.528	2.217	0.427	1.442	0.228										
5	0.000	1.317	0.337	0.923	0.110										
6	0.476	1.855	0.907	0.275	0.805										
7	0.178	3.254	0.585	0.000	0.977										
8	0.131	2.836	2.318	0.571	0.532	0.207	1.293	0.456	0.178	0.314	0.008	1.271	0.231	0.191	0.008
9	2.758	4.182	1.903	1.529	0.756	0.586	2.143	0.786	1.062	0.393	0.387	2.121	0.560	1.076	0.387
10	0.172	2.048	0.447	0.263	0.959	0.521	2.064	1.082	-0.004	1.052	0.322	2.042	0.857	0.01	0.322
11	2.455	4.371	1.433	0.732	0.759	0.901	2.238	1.380	0.677	0.591	0.225	2.163	0.613	0.722	0.225

The results of the correlation analysis of the resolution matrix of successive chromatographic peaks are presented in Table 11. The values and associated significances of the Pearson and Spearman correlation coefficients sustained the linearity of the relationship between experimental and estimated values. The analysis of the results presented in Table 11 revealed that for both equations the Pearson correlation coefficient is greater than the Spearman correlation coefficient, leading to the conclusion that the investigated response function is a quantitative variable. With a single exceptions (Gamma correlation analysis), all correlation method sustained the validity of the model from Eq (1.1) and Eq (1.2) (p ≤ 5.54·10^-3^). With one exception (Kendall τ_c_, τ_Ken,c_ = 0.4900), the values of correlation coefficient were higher than 0.5, indicating moderate to good correlations between experimental and estimated values. Four out of seven correlation coefficients (Kendall τa, Kendall τb, Kendall τc, and Gamma) had values less than or equal with 0.4737, indicating a weak correlation between experimental and estimated by Eq (1.2) values. There could not be identified any statistically significant differences between correlation coefficients presented in Table 11. The lower p-values (0.2348 - Eq (1.1), and 0.2592 - Eq (1.2)) were obtained when Pearson and Kendall τc correlation coefficients were compared.

**Table 11:** Results of correlation analysis: resolution matrix of successive chromatographic peaks (experimental vs estimated).

Name	Correlation coefficient	p-value	Statistical parameter
**Estimated by Eq(1.1), n = 20**			
Pearson	r = 0.7446	1.66·10^-4^	t_Prs,1_ = 4.73
Spearman	ρ = 0.6692	1.25·10^-3^	t_Spm,1_ = 3.82
Semi-Q	r_sQ_ = 0.7059	5.06·10^-4^	t_sQ_ = 4.23
Kendall τa	τ_Ken,a_ = 0.5158	1.47·10^-3^	Z_Ken,τa_ = 3.18
Kendall τb	τ_Ken,b_ = 0.5158	1.47·10^-3^	Z_Ken,τb_ = 3.18
Kendall τc	τ_Ken,c_ = 0.4900	2.52·10^-3^	Z_Ken,τc_ = 3.02
Gamma	Γ = 0.5158	1.01·10^-1^	Z_Γ_ = 1.64
**Estimated by Eq(1.2), n = 25**			
Pearson	r = 0.6821	9.24·10^-4^	t_Prs,1_ = 3.96
Spearman	ρ = 0.6361	2.57·10^-3^	t_Spm,1_ = 3.50
Semi-Q	r_sQ_ = 0.6587	1.59·10^-3^	t_sQ_ = 3.71
Kendall τa	τ_Ken,a_ = 0.4737	3.50·10^-3^	Z_Ken,τa_ = 2.92
Kendall τb	τ_Ken,b_ = 0.4737	3.50·10^-3^	Z_Ken,τb_ = 2.92
Kendall τc	τ_Ken,c_ = 0.4500	5.54·10^-3^	Z_Ken,τc_ = 2.77
Gamma	Γ = 0.4737	1.67·10^-1^	Z_Γ_ = 1.38

Seven global indicators of separation were introduced and calculated (see Table 2):

÷ The number distinct compounds on chromatogram DCN - Eq (7);
÷ The string of standard deviation of retardation factors ordered ascending and estimated by Eq (1) compared with ideal positions of the peaks obtained through experiment RFD - Eq (9);
÷ The string of sum of the peak resolutions obtained through experiment RSS - Eq (12);
÷ The squared of effective plate number QN- Eq (13);
÷ Average peaks separation (into experiment) RSA - Eq (15);
÷ The string of mean resolution calculated with Minkowski experimental peaks RSR - Eq (17);
÷ The string of experimental peaks with minimal resolution QF- Eq (18).

The values associated to the global indicators of separation are presented in Table 12 and Table 13, respectively. The results of correlation analysis are presented in Table 14 and Table 15, respectively.

In investigation of the number of distinct compounds on chromatogram, no difference was obtained by both models (Eq (1.1), and Eq (1.2), respectively) for the experiments from 9 to 11 (integer numbers were considered). The same difference of two was obtained between experimental and estimated by both models for experiment no. 8; a difference of 1 for experiment no. 1 when Eq (1.2) was investigated.

Regarding the string of standard deviation of retardation factors ordered ascending and estimated by Eq (1.1) compared with the ideal positions of the peaks obtained through experiment, the lowest difference between experimental and estimated value is obtained by experiment no. 11 (0.001, Eq (1.1)), while the higher difference by the experiment no. 8 (-0.114, Eq (1.1)). The lowest difference of -0.002 was obtained by experiments nos. 8,9 & 10 and the largest difference of -0.010 was obtained by experiment no. 1 when the Eq (1.2) was investigated.

**Table 12:** Four (response functions) global indicators on chromatography: experimental vs estimated.

No.	Experimental				Estimated by Eq(1.1), n = 4				Estimated by Eq(1.2), n = 5			
	DCN	RFD	RSS	QN	DCN	RFD	RSS	QN	DCN	RFD	RSS	QN
1	5	0.047	5.730	71.900					4 (4.222)	0.057	3.73	83.857
2	1	0.283	0.000	114.680								
3	4	0.081	2.260	108.700								
4	5	0.055	4.610	89.550								
5	4	0.078	2.580	103.840								
6	5	0.057	3.510	83.790								
7	3	0.097	4.020	79.280								
8	4	0.067	5.860	59.460	2 (2.368)	0.181	2.135	98.554	2 (2.200)	0.183	1.703	101.14
9	5	0.036	10.370	71.240	5 (5.168)	0.042	4.574	85.744	5 (5.000)	0.044	4.142	88.326
10	4	0.076	2.930	80.920	4 (4.328)	0.062	3.661	89.591	4 (4.160)	0.064	3.229	92.174
11	5	0.040	8.990	63.380	5 (5.220)	0.039	5.192	79.123	5 (4.650)	0.046	3.725	87.895
DCN = number of distinct compounds on chromatogram; RFD = string of standard deviation of retardation factors ordered ascending and estimated by Eq(1.1) and Eq(1.2), respectively compared with ideal positions of the peaks obtained through experiment; RSS = string of sum of the peak resolutions obtained through experiment; QN = squared of effective plate number.												

The analysis of the string of sum of the peak resolutions revealed a difference between experimental and estimated of -0.731 (experiment no. 10, Eq (1.1)) and of 5.795 (experiment no. 9, Eq (1.1)). The investigation of Eq (1.2) revealed that the lowest difference of 0.432 was obtained by experiments 8, 9 & 10, and the largest of 2.000 by experiment no. 1. The lowest difference for the squared of effective plate number response function was obtained in the experiments 8, 9 & 10 (-2.582, Eq (1.2)) and the highest one by the experiment no. 8 (-39.094, Eq (1.2)).

**Table 13:** Other three global indicators of chromatographic parameters: experimental vs estimated.

No	Experimental			Estimated by Eq(1.1), n = 4			Estimated by Eq(1.2), n = 5		
	RSA	RSR	QF	RSA	RSR	QF	RSA	RSR	QF
1	1.434	1.285	0.310				0.932	0.636	0.075
2	0.000	0.000	0.000						
3	0.566	0.401	0.000						
4	1.153	1.035	0.427						
5	0.644	0.452	0.000						
6	0.878	0.778	0.275						
7	1.004	0.559	0.000						
8	1.464	1.169	0.131	0.533	0.380	0.028	0.425	0.240	0.000
9	2.593	2.498	1.529	1.144	1.021	0.344	1.035	0.881	0.293
10	0.733	0.573	0.172	0.916	0.696	0.105	0.807	0.556	0.054
11	2.248	2.038	0.732	1.299	1.180	0.311	0.931	0.704	0.138
RSA = average peaks separation (into experiment); RSR = string of mean resolution calculated with Minkowski of experimental peaks; QF = string of experimental peaks with minimal resolution.									

The last three global response functions had the same difference pattern between experimental and estimated values. The lowest difference is obtained in experiment no. 10 and the highest difference by experiment no. 9 for Eq (1.1) and by experiment no. 8, and experiment no. 11, respectively, for Eq (1.2).

The correlation analysis was applied also on the global quality factors. The lower sample size of experimental data is the major limitation of this analysis and explained the absence of the significance (p ≥ 0.05).

Other three response functions were implemented and computed resolution divided by the number of effective plates - RSP; informationa energy - IEne; and informational entropy - IEnt. The experimental an estimated values are presented in Table 16.

The results of correlation analysis on these response functions are presented in Table 17 and Table 18.

The difference of resolution divided by the number of effective plates obtained experimentally and estimated by model from Eq (1.1) varied into a large range: from -3.176 for experiment no. 10 to 36.548 for experiment no. 9 (see Table 15). The variation of the informational energy and informational entropy varied on the same pattern: the lowest difference between experimental and estimated values was obtained by experiment no. 11, while the highest values were obtained by the experiment no. 8 - Eq (1.1).

All response functions presented in Table 16 varied by the same pattern for Eq (1.2): the lowest difference is obtained by experiment no. 8 and the highest one by the experiment no. 1.

Informational response functions, energy and entropy, are parameters that investigated the disorder into the system (in our case the disorders into chromatographic analysis). For both response functions, values lower than 0.41 were obtained, these values being not statistically significant.

**Table 14:** Results of correlation analysis on global quality factors: Eq (1.1), n = 4.

Name	Correlation coefficient	p-value	Statistical parameter	Name	Correlation coefficient	p-value	Statistical parameter
**DCN**				**RSA**			
Pearson	r = 0.8165	1.80·10^-1^	t_Prs,1_ = 2.0	Pearson	r = 0.5905	4.09·10^-1^	t_Prs,1_ = 1.03
Spearman	ρ = 0.9428	5.72·10^-2^	t_Spm,1_ = 4.0	Spearman	ρ = 0.6000	4.00·10^-1^	t_Spm,1_ = 1.06
Semi-Q	r_sQ_ = 0.8457	1.23·10^-1^	t_sQ_ = 2.59	Semi-Q	r_sQ_ = 0.5952	4.05·10^-1^	t_sQ_ = 1.05
Kendall tau-a	τ_Ken,a_ = 0.6667	1.75·10^-1^	Z_Ken,τa_ = 1.36	Kendall tau-a	τ_Ken,a_ = 0.3333	4.97·10^-1^	Z_Ken,τa_ = 0.68
Kendall tau-b	τ_Ken,b_ = 0.7303	1.49·10^-1^	Z_Ken,τb_ = 1.44	Kendall tau-b	τ_Ken,b_ = 0.3333	4.97·10^-1^	Z_Ken,τb_ = 0.68
Kendall tau-c	τ_Ken,c_ = 0.5000	2.79·10^-1^	Z_Ken,τc_ = 1.08	Kendall tau-c	τ_Ken,c_ = 0.2500	6.10·10^-1^	Z_Ken,τc_ = 0.51
Gamma	Γ = 1.0000	4.15·10^-2^	Z_Γ_ = 2.04	Gamma	Γ = 0.3333	8.21·10^-1^	Z_Γ_ = 0.23
**RFD**				**RSR**			
Pearson	r = 0.5434	4.56·10^-1^	t_Prs,1_ = 0.92	Pearson	r = 0.7118	2.88·10^-1^	t_Prs,1_ = 2.05
Spearman	ρ = 0.6000	4.00·10^-1^	t_Spm,1_ = 1.06	Spearman	ρ = 0.6000	3.46·10^-1^	t_Spm,1_ = 1.06
Semi-Q	r_sQ_ = 0.5710	4.29·10^-1^	t_sQ_ = 0.98	Semi-Q	r_sQ_ = 0.6535	5.73·10^-1^	t_sQ_ = 1.22
Kendall tau-a	τ_Ken,a_ = 0.3333	4.97·10^-1^	Z_Ken,τa_ = 0.68	Kendall tau-a	τ_Ken,a_ = 0.3333	4.47·10^-1^	Z_Ken,τa_ = 0.68
Kendall tau-b	τ_Ken,b_ = 0.3333	4.97·10^-1^	Z_Ken,τb_ = 0.68	Kendall tau-b	τ_Ken,b_ = 0.3333	4.97·10^-1^	Z_Ken,τb_ = 0.68
Kendall tau-c	τ_Ken,c_ = 0.250	6.10·10^-1^	Z_Ken,τc_ = 0.51	Kendall tau-c	τ_Ken,c_ = 0.2500	6.10·10^-1^	Z_Ken,τc_ = 0.51
Gamma	Γ = 0.3333	8.21·10^-1^	Z_Γ_ = 0.23	Gamma	Γ = 0.3333	8.21·10^-1^	Z_Γ_ = 0.23
**RSS**				**QF**			
Pearson	r = 0.5906	4.09·10^-1^	t_Prs,1_ = 1.04	Pearson	r = 0.8936	1.06·10^-1^	t_Prs,1_ = 2.82
Spearman	ρ = 0.6000	4.00·10^-1^	t_Spm,1_ = 1.13	Spearman	ρ = 1.0000	5.47·10^-2^	t_Spm,1_ = 4.10
Semi-Q	r_sQ_ = 0.5953	4.05·10^-1^	t_sQ_ = 1.05	Semi-Q	r_sQ_ = 0.9453	6.68·10^-2^	t_sQ_ = 2.82
Kendall	τ_Ken,a_ = 0.3333	1.97·10^-1^	Z_Ken,τa_ = 0.68	Kendall	τ_Ken,a_ = 1.0000	4.15·10^-2^	Z_Ken,τa_ = 2.04
Kendall	τ_Ken,b_ = 0.3333	4.97·10^-1^	Z_Ken,τb_ = 0.68	Kendall	τ_Ken,b_ = 1.0000	4.15·10^-2^	Z_Ken,τb_ = 2.04
Kendall	τ_Ken,c_ = 0.2500	6.10·10^-1^	Z_Ken,τc_ = 0.51	Kendall	τ_Ken,c_ = 0.7500	1.26·10^-1^	Z_Ken,τc_ = 1.53
Gamma	Γ = 0.3333	8.21·10^-1^	Z_Γ_ = 0.23	Gamma	Γ = 1.0000	4.15·10^-2^	Z_Γ_ = 2.04
**QN**							
Pearson	r = -0.1588	9.85·10^-1^	t_Prs,1_ = 0.22	n = sample size; DCN = number of distinct compounds on chromatogram; RFD = string of standard deviation of retardation factors estimated by Eq(1) ordered ascending compared with ideal positions of the peaks obtained through experiment; RSS = string of sum of the peak resolutions obtained through experiment; QN = squared of effective plate number;RSA = average peaks separation (into experiment); RSR = string of Minkowski mean resolution of experimental peaks; QF = string of experimental peaks with minimal resolution.			
Spearman	ρ = -0.2000	8.22·10^-1^	t_Spm,1_ = 0.29				
Semi-Q	r_sQ_ = 0.1782	8.00·10^-1^	t_sQ_ = 0.25				
Kendall tau-a	τ_Ken,a_ = 0.0000	1.00	Z_Ken,τa_ = 0.00				
Kendall tau-b	τ_Ken,b_ = 0.0000	1.00	Z_Ken,τb_ = 0.00				
Kendall tau-c	τ_Ken,c_ = 0.0000	1.00	Z_Ken,τc_ = 0.00				
Gamma	Γ = 0.0000	1.00	Z_Γ_ = 0.00				

**Table 15:** Results of correlation analysis on global quality factors: Eq (1.2), n = 5.

Name	Correlation coefficient	p-value	Statistical parameter	Name	Correlation coefficient	p-value	Statistical parameter
**DCN**				**RSA**			
Pearson	r = 0.7454	1.48·10^-1^	t_Prs,1_ = 3.75	Pearson	r = 0.4698	4.25·10^-1^	t_Prs,1_ = 0.92
Spearman	ρ = 0.4722	4.22·10^-1^	t_Spm,1_ = 0.93	Spearman	ρ = 0.5000	3.91·10^-1^	t_Spm,1_ = 1.00
Semi-Q	r_sQ_ = 0.5933	2.92·10^-1^	t_sQ_ = 1.28	Semi-Q	r_sQ_ = 0.4847	4.08·10^-1^	t_sQ_ = 0.96
Kendall tau-a	τ_Ken,a_ = 0.3000	4.62·10^-1^	Z_Ken,τa_ = 0.73	Kendall tau-a	τ_Ken,a_ = 0.4000	3.27·10^-1^	Z_Ken,τa_ = 0.98
Kendall tau-b	τ_Ken,b_ = 0.3162	4.49·10^-1^	Z_Ken,τb_ = 0.76	Kendall tau-b	τ_Ken,b_ = 0.4000	3.27·10^-1^	Z_Ken,τb_ = 0.98
Kendall tau-c	τ_Ken,c_ = 0.2400	5.44·10^-1^	Z_Ken,τc_ = 0.61	Kendall tau-c	τ_Ken,c_ = 0.3200	4.33·10^-1^	Z_Ken,τc_ = 0.78
Gamma	Γ = 0.4286	6.53·10^-1^	Z_Γ_ = 0.45	Gamma	Γ = 0.4000	6.95·10^-1^	Z_Γ_ = 0.39
**RFD**				**RSR**			
Pearson	r = 0.5520	3.35·10^-1^	t_Prs,1_ = 1.15	Pearson	r = 0.6827	2.04·10^-1^	t_Prs,1_ = 2.62
Spearman	ρ = 0.9000	3.74·10^-2^	t_Spm,1_ = 3.58	Spearman	ρ = 0.9000	3.74·10^-2^	t_Spm,1_ = 3.58
Semi-Q	r_sQ_ = 0.7049	1.84·10^-1^	t_sQ_ = 1.72	Semi-Q	r_sQ_ = 0.7838	1.17·10^-1^	t_sQ_ = 2.19
Kendall tau-a	τ_Ken,a_ = 0.8000	5.00·10^-2^	Z_Ken,τa_ = 1.96	Kendall tau-a	τ_Ken,a_ = 0.8000	5.00·10^-2^	Z_Ken,τa_ = 1.96
Kendall tau-b	τ_Ken,b_ = 0.8000	5.00·10^-2^	Z_Ken,τb_ = 1.96	Kendall tau-b	τ_Ken,b_ = 0.8000	5.00·10^-2^	Z_Ken,τb_ = 1.96
Kendall tau-c	τ_Ken,c_ = 0.6400	1.17·10^-1^	Z_Ken,τc_ = 1.57	Kendall tau-c	τ_Ken,c_ = 0.6400	1.17·10^-1^	Z_Ken,τc_ = 1.57
Gamma	Γ = 0.8000	1.17·10^-1^	Z_Γ_ = 1.57	Gamma	Γ = 0.8000	1.17·10^-1^	Z_Γ_ = 1.57
**RSS**				**QF**			
Pearson	r = 0.4691	4.25·10^-1^	t_Prs,1_ = 0.92	Pearson	r = 0.9871	1.76·10^-3^	t_Prs,1_ = 10.67
Spearman	ρ = 0.5000	3.91·10^-1^	t_Spm,1_ = 1.00	Spearman	ρ = 1.0000	1.24·10^-2^	t_Spm,1_ = 5.41
Semi-Q	r_sQ_ = 0.4843	4.08·10^-1^	t_sQ_ = 0.96	Semi-Q	r_sQ_ = 0.9935	6.26·10^-4^	t_sQ_ = 15.14
Kendall	τ_Ken,a_ = 0.4000	3.27·10^-1^	Z_Ken,τa_ = 0.98	Kendall	τ_Ken,a_ = 1.0000	1.43·10^-2^	Z_Ken,τa_ = 2.45
Kendall	τ_Ken,b_ = 0.4000	3.27·10^-1^	Z_Ken,τb_ = 0.98	Kendall	τ_Ken,b_ = 1.0000	1.43·10^-2^	Z_Ken,τb_ = 2.45
Kendall	τ_Ken,c_ = 0.3200	4.33·10^-1^	Z_Ken,τc_ = 0.78	Kendall	τ_Ken,c_ = 0.8000	5.00·10^-2^	Z_Ken,τc_ = 1.96
Gamma	Γ = 0.4000	6.95·10^-1^	Z_Γ_ = 0.39	Gamma	Γ = 1.0000	1.43·10^-2^	Z_Γ_ = 2.45
**QN**							
Pearson	r = -0.4189	4.82·10^-1^	t_Prs,1_ = 0.80	n = sample size; DCN = number of distinct compounds on chromatogram; RFD = string of standard deviation of retardation factors ordered ascending and estimated by Eq(1) compared with ideal positions of the peaks obtained through experiment; RSS = string of sum of the peak resolutions obtained through experiment; QN = squared of effective plate number; RSA = average peaks separation (into experiment); RSR = string of mean resolution calculated with Minkowski experimental peaks; QF = string of experimental peaks with minimal resolution.			
Spearman	ρ = -0.3000	6.24·10^-1^	t_Spm,1_ = 0.54				
Semi-Q	r_sQ_ = 0.3545	5.58·10^-1^	t_sQ_ = 0.66				
Kendall tau-a	τ_Ken,a_ = 0.2000	6.24·10^-1^	Z_Ken,τa_ = 0.49				
Kendall tau-b	τ_Ken,b_ = 0.2000	6.24·10^-1^	Z_Ken,τb_ = 0.49				
Kendall tau-c	τ_Ken,c_ = 0.1600	7.05·10^-1^	Z_Ken,τc_ = 0.39				
Gamma	Γ = 0.2000	9.22·10^-1^	Z_Γ_ = 0.10				

**Table 16:** Resolution ratio, informational energy and entropy: experimental vs estimated.

No.	Experimental			Estimated by Eq(1.1), n = 4			Estimated by Eq(1.2), n = 5		
	RSP	IEnt	IEne	RSP	IEnt	IEne	RSP	IEnt	IEne
1	31.900	4.000	16.000				17.678	10.407	2.67
2	0.000	11.610	0.000						
3	8.300	11.610	0.000						
4	20.600	8.000	8.000						
5	9.900	11.610	0.000						
6	16.800	8.000	8.000						
7	20.300	11.610	0.000						
8	39.400	4.000	16.000	11.096	10.371	2.560	8.024	11.754	0.00
9	58.200	2.000	18.000	21.652	7.338	9.280	18.58	8.722	6.40
10	14.500	11.610	0.000	17.676	9.360	4.800	14.604	10.744	1.92
11	56.700	4.750	14.000	27.285	5.203	13.565	16.852	9.902	3.78
RSP = resolution divided by the number of effective plates; IEnt = informational energy; IEne = informational entropy.									

**Table 17:** Results of correlation analysis for response functions presented in Table 15: Eq (1.1), n = 4.

Name	Correlation coefficient	p-value		Statistical parameter	Name	Correlation coefficient	p-value	Statistical parameter
**RSP**					**IEnt**			
Pearson	r = 0.5326		4.67·10^-1^	t_Prs,1_ = 0.90	Pearson	r = 0.3188	6.81·10^-1^	t_Prs,1_ = 0.48
Spearman	ρ = 0.6000		4.00·10^-1^	t_Spm,1_ = 1.06	Spearman	ρ = 0.0000	1.00	t_Spm,1_ = 0.00
Semi-Q	r_sQ_ = 0.5653		4.35·10^-1^	t_sQ_ = 0.97	Semi-Q	r_sQ_ = 0.0000	1.00	t_sQ_ = 0.00
Kendall tau-a	τ_Ken,a_ = 0.3333		4.97·10^-1^	Z_Ken,τa_ = 0.68	Kendall tau-a	τ_Ken,a_ = 0.0000	1.00	Z_Ken,τa_ = 0.00
Kendall tau-b	τ_Ken,b_ = 0.3333		4.97·10^-1^	Z_Ken,τb_ = 0.68	Kendall tau-b	τ_Ken,b_ = 0.0000	1.00	Z_Ken,τb_ = 0.00
Kendall tau-c	τ_Ken,c_ = 0.2500		6.10·10^-1^	Z_Ken,τc_ = 0.51	Kendall tau-c	τ_Ken,c_ = 0.0000	1.00	Z_Ken,τc_ = 0.00
Gamma	Γ = 0.3333		8.21·10^-1^	Z_Γ_ = 0.23	Gamma	Γ = 0.0000	1.00	Z_Γ_ = 0.00
**IEne**								
Pearson	r = 0.2962		7.04·10^-1^	t_Prs,1_ = 0.44	RSP = resolution divided by the number of effective plates; IEnt = informational energy; IEne = informational entropy; n = sample size.			
Spearman	ρ = 0.0000		1.00	t_Spm,1_ = 0.00				
Semi-Q	r_sQ_ = 0.0000		1.00	t_sQ_ = 0.00				
Kendall	τ_Ken,a_ = 0.0000		1.00	Z_Ken,τa_ = 0.00				
Kendall	τ_Ken,b_ = 0.0000		1.00	Z_Ken,τb_ = 0.00				
Kendall	τ_Ken,c_ = 0.0000		1.00	Z_Ken,τc_ = 0.00				
Gamma	Γ = 0.0000		1.00	Z_Γ_ = 0.00				

**Table 18:** Results of correlation analysis for response functions presented in Table 15: Eq (1.2), n = 5.

Name	Correlation coefficient	p-value	Statistical parameter	Name	Correlation coefficient	p-value	Statistical parameter
**RSP**				**IEnt**			
Pearson	r = 0.2864	6.40·10^-1^	t_Prs,1_ = 0.52	Pearson	r = 0.3770	5.32·10^-1^	t_Prs,1_ = 0.71
Spearman	ρ = 0.5000	3.91·10^-1^	t_Spm,1_ = 1.00	Spearman	ρ = 0.4104	4.92·10^-1^	t_Spm,1_ = 0.78
Semi-Q	r_sQ_ = 0.3784	5.30·10^-1^	t_sQ_ = 0.71	Semi-Q	r_sQ_ = 0.3934	5.12·10^-1^	t_sQ_ = 0.74
Kendall tau-a	τ_Ken,a_ = 0.4000	3.27·10^-1^	Z_Ken,τa_ = 0.98	Kendall tau-a	τ_Ken,a_ = 0.3000	4.62·10^-1^	Z_Ken,τa_ = 0.73
Kendall tau-b	τ_Ken,b_ = 0.4000	3.27·10^-1^	Z_Ken,τb_ = 0.98	Kendall tau-b	τ_Ken,b_ = 0.3162	4.48·10^-1^	Z_Ken,τb_ = 0.76
Kendall tau-c	τ_Ken,c_ = 0.3200	4.33·10^-1^	Z_Ken,τc_ = 0.78	Kendall tau-c	τ_Ken,c_ = 0.2400	5.44·10^-1^	Z_Ken,τc_ = 0.61
Gamma	Γ = 0.4000	6.95·10^-1^	Z_Γ_ = 0.39	Gamma	Γ = 0.3333	7.85·10^-1^	Z_Γ_ = 0.27
**IEne**							
Pearson	r = 0.3152	6.05·10^-1^	t_Prs,1_ = 0.58	RSP = resolution divided by the number of effective plates; IEnt = informational energy; IEne = informational entropy; n = sample size.			
Spearman	ρ = 0.4104	4.92·10^-1^	t_Spm,1_ = 0.78				
Semi-Q	r_sQ_ = 0.3596	5.52·10^-1^	t_sQ_ = 0.67				
Kendall	τ_Ken,a_ = 0.3000	4.62·10^-1^	Z_Ken,τa_ = 0.73				
Kendall	τ_Ken,b_ = 0.3162	4.48·10^-1^	Z_Ken,τb_ = 0.76				
Kendall	τ_Ken,c_ = 0.2400	5.44·10^-1^	Z_Ken,τc_ = 0.61				
Gamma	Γ = 0.3333	7.85·10^-1^	Z_Γ_ = 0.27				

The summary of the acceptance of the linear relationships between experimental and estimated values by the Eq (1.1) and Eq (1.2) for the investigated response functions is presented in Table 19.

### Experiments Quality Assessment

RF, RFO, RSM, RSO, and QF chromatographic response functions can be accepted as being dependent on mobile phase composition with a good confidence, according to the results presented in Table 19. This partial conclusion can be used now backward, in order to see what is wrong (if there is something) in the experiments. Table 20 presented the biggest 20% differences between experimental and predicted by the models values. The Diff column from Table 20 contains relative differences calculated as follows:


Diff = 50·|Exp-Est|/(Exp+Est) [%]
(18)

Results from Table 20 can be analyzed in terms of relative deviation using Eq (18) if the values of relative deviation obtained for a given model and chromatographic response functions are ordered by rank.

**Table 19:** Summary of validation the response functions estimated by the model from Eq (1.1) and Eq (1.2), respectively.

Parameter	Pearson	Spearman	Semi-Q	Kendall τa	Kendall τb	Kendall τc	Gamma
**Eq (1.1)**							
RF	✓	✓	✓	✓	✓	✓	✓
RFO	✓	✓	✓	✓	✓	✓	✓
RSM	✓	✓	✓	✓	✓	✓	✕
RSO	✓	✓	✓	✓	✓	✓	✕
QF	✕	✕	✕	✓	✓	✕	✓
DCN	✕	✕	✕	✕	✕	✕	✓
RFD	✕	✕	✕	✕	✕	✕	✕
RSS	✕	✕	✕	✕	✕	✕	✕
QN	✕	✕	✕	✕	✕	✕	✕
RSA	✕	✕	✕	✕	✕	✕	✕
RSR	✕	✕	✕	✕	✕	✕	✕
RSP	✕	✕	✕	✕	✕	✕	✕
IEne	✕	✕	✕	✕	✕	✕	✕
IEnt	✕	✕	✕	✕	✕	✕	✕
**Eq (1.2)**							
RF	✓	✓	✓	✓	✓	✓	✓
RFO	✓	✓	✓	✓	✓	✓	✓
RSM	✓	✓	✓	✓	✓	✓	✓
RSO	✓	✓	✓	✓	✓	✓	✕
QF	✓	✓	✓	✓	✓	✓	✓
DCN	✕	✕	✕	✕	✕	✕	✕
RFD	✕	✓	✕	✓	✓	✕	✕
RSS	✕	✕	✕	✕	✕	✕	✕
QN	✕	✕	✕	✕	✕	✕	✕
RSA	✕	✕	✕	✕	✕	✕	✕
RSR	✕	✓	✕	✓	✓	✕	✕
RSP	✕	✕	✕	✕	✕	✕	✕
IEne	✕	✕	✕	✕	✕	✕	✕
IEnt	✕	✕	✕	✕	✕	✕	✕
✓ = statistical significant at a significance level of 5%; ✕ = statistical insignificant at a significance level of 5%; RF = retardation factor; RFO = retardation factor ordered ascending by the chromatographic peak; RSM = resolution of pairs of compounds; RSO = resolution matrix of successive chromatographic peaks; DCN = number of distinct compounds on chromatogram; RFD = string of standard deviation of retardation factors ordered ascending and estimated by Eq(1.1), Eq(1.2) respectively, compared with ideal positions of the peaks obtained through experiment; RSS = string of sum of the peak resolutions obtained through experiment; QN = squared of effective plate number; RSA = average peaks separation (into experiment); RSR = string of mean resolution calculated with Minkowski experimental peaks; QF = string of experimental peaks with minimal resolution; RSP = resolution divided by the number of effective plates; IEnt = informational energy; IEne = informational entropy;							

**Table 20:** Summary of validation the response functions estimated by the model from Eq (1.1) and Eq (1.2), respectively.

No	Model	CRF	Estimated	Experimental	Difference (%)	Group Rank	Exp No
1	Eq (1.1)	RF	0.505	0.181	23.62	1	11
2	Eq (1.1)	RF	0.253	0.709	23.70	2	11
3	Eq (1.1)	RF	0.216	0.072	25.00	3	8
4	Eq (1.1)	RF	0.203	0.060	27.19	4	8
5	Eq (1.1)	RSM	0.456	2.318	33.56	1	8
6	Eq (1.1)	RSM	1.38	7.191	33.90	2	11
7	Eq (1.1)	RSM	8.665	1.433	35.81	3	11
8	Eq (1.1)	RSM	0.677	4.371	36.59	4	11
9	Eq (1.1)	RSM	-0.004	0.263	51.54	5	10
10	Eq (1.1)	RFO	0.393	0.756	15.80	1	9
11	Eq (1.1)	RFO	0.347	0.683	16.31	2	9
12	Eq (1.1)	RFO	0.219	0.072	25.26	3	8
13	Eq (1.1)	RFO	0.200	0.06	26.92	4	8
14	Eq (1.1)	RSO	0.586	2.758	32.48	1	9
15	Eq (1.1)	RSO	0.456	2.318	33.56	2	8
16	Eq (1.1)	RSO	-0.004	0.263	51.54	3	10
18	Eq (1.1)	QF	0.028	0.131	32.39	1	8
19	Eq (1.2)	RF	0.311	0.709	19.51	1	11
20	Eq (1.2)	RF	0.322	0.759	20.21	2	11
21	Eq (1.2)	RF	0.438	0.181	20.76	3	11
22	Eq (1.2)	RF	0.215	0.072	24.91	4	8
23	Eq (1.2)	RF	0.220	0.060	28.57	5	8
24	Eq (1.2)	RSM	2.646	14.473	34.54	1	8
25	Eq (1.2)	RSM	1.819	10.234	34.91	2	11
26	Eq (1.2)	RSM	0.196	1.118	35.08	3	1
27	Eq (1.2)	RSM	0.722	4.371	35.82	4	11
28	Eq (1.2)	RSM	0.387	2.758	37.69	5	9
29	Eq (1.2)	RSM	0.325	2.392	38.04	6	8
30	Eq (1.2)	RSM	0.231	2.318	40.94	7	8
31	Eq(1.2)	RSM	0.613	7.191	42.15	8	11
32	Eq (1.2)	RSM	0.008	0.131	44.24	9	8
33	Eq (1.2)	RSM	0.010	0.263	46.34	10	10
34	Eq (1.2)	RFO	0.289	0.573	16.47	1	9
35	Eq (1.2)	RFO	0.364	0.756	17.50	2	9
36	Eq (1.2)	RFO	0.317	0.683	18.30	3	9
37	Eq (1.2)	RFO	0.219	0.072	25.26	4	8
38	Eq (1.2)	RFO	0.215	0.060	28.18	5	8
39	Eq (1.2)	RSO	0.231	2.318	40.94	1	8
40	Eq (1.2)	RSO	0.225	2.455	41.60	2	11
41	Eq (1.2)	RSO	0.008	0.131	44.24	3	8
42	Eq (1.2)	RSO	0.010	0.263	46.34	4	10
43	Eq (1.2)	QF	-0.023	0.131	71.30	1	8

**Figure 1:**
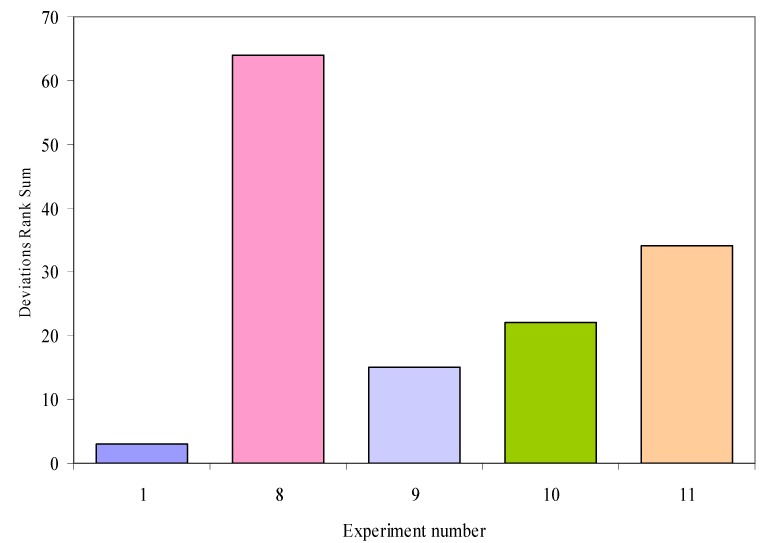
Rank sums for relative deviations of investigated experiments.

A qualitative measure of experiments results can be obtained by constructing the graphical representation of experiments based on the rank sums of relative deviations. Figure 1 presents the plot of rank sums deviations for the experiments included in the estimation.

The analysis of values presented in Figure 1 showed that over 60 relative deviation ranks sum are recorded for experiment no. 8 (about 46.4%), followed by over 30 relative deviation ranks sum for experiment no. 11 (about 24.6%, about half relative to the experiment no. 8). Considering a normal distribution of unbiased error, the expected distribution of deviations rank sum is uniform. Thus, the expected frequencies for ranks sums in investigated experiments are no more than 25% (no more than 34 relative deviation ranks sum). Concluding, the results obtained for deviations of rank sums suggest that results from experiment no. 8 are questionable.

## Conclusions

The analyses of the data presented in Table 19, Table 20 and Figure 1 leads to the following conclusions:

1. The model presented in Eq (1.2) seems to be more reliable for the estimation of chromatographic response functions on investigated androstane isomers. Four response functions (RF - retardation factor; RFO - retardation factor ordered ascending by the chromatographic peak; RSM - resolution of pairs of compounds; QF - string of experimental peaks with minimal resolution) revealed statistically significant linear relationships between experimental and estimated values.
2. The models presented in Eq (1.1) is valid and reliable in investigation of retardation factor, retardation factor ordered ascending by the chromatographic response, resolution of pairs of compounds and resolution matrix of successive chromatographic peaks;
3. Good performances are obtained in estimation of resolution of pairs of compounds but the relationship between experimental and estimated values by Eq (1.1) and Eq (1.2) could be questionable due to the absence of significantly statistic Gamma correlation coefficient;
4. Some estimation abilities were observed in investigation of the string of standard deviation of retardation factors ordered ascending estimated by Eq (1.2) compared with ideal positions of the peaks obtained through experiment; and of the string of Minkowski type mean resolution calculated by Eq (1.2) with experimental peaks. These two chromatographic response functions seem to be qualitative and rank variables.
5. Two global response functions for the separation, abbreviated as QF and DCN recorded a weak acceptance in investigation of Eq (1.1). Thus, QF are rejected at 95% confidence by Spearman (with 5.47% error), Semi-Q (6.68% error), Pearson (with 10.6% error) and Kendall τ_c_ (with 12.6% error) even if the correlations are good (over 0.75). The small dimension of the sample size, not grater enough to provide statistical significance of the obtained correlations, explained with a good confidence the rejection of these correlations. Note that QF chromatographic response function is in fact a minimum function of resolutions of the separation, resolutions that are accepted by the model from Eq (1.1) - see 2^nd^ conclusion. DCN is statistically significant by the Goodman-Kruskal method (concordant vs. discordant) and is near to be statistically significant by the Spearman method (5.72% error). Thus, the rejection is recorded for a quantitative correlation, but a possible acceptance is seen by the qualitative correlation. Again, small sample size is against of a solid statistical conclusion for DCN.
6. The results presented in Experiments Quality Assessment subsection sustain the hypothesis that the proposed equations (Eq (1.1) and Eq (1.2), respectively) could be used in order to verify the quality of experimental data. The results obtained for deviations of rank sums suggest that the experimental data of the experiment no. 8 are questionable.

## References

[1] Senchenkova E.M., Gillispie Ch. C. (1976). Tsvet (or Tswett). Mikhail Semenovich (1872 - 1919). Dictionary of scientific biography.

[2] Tswett M. (1906). Adsorption analysis and chromatographic method. Application to the chemistry of the chlorophyll. Ber. Deut. Bot. Ges.

[3] Duarte A.C., Capelo S. (2006). Application of chemometrics in separation science. J. Liq. Chrom. Rel. Technol.

[4] Xu L., Tang L.-J., Cai C.-B., Wu H.-L., Shen G.-L., Yu R.-Q., Jiang J.-H. (2008). Chemometric methods for evaluation of chromatographic separation quality from two-way data-A review. Anal. Chim. Acta.

[5] Miyake T., Yafuso M. (2005). Pollination of Alocasia cucullata (Araceae) by two Colocasiomyia flies known to be specific pollinators for Alocasia odora. Plant Species Biol.

[6] De Abreu I.N., Sawaya A.C., Eberlin M.N., Mazzafera P. (2005). Production of pilocarpine in callus of jaborandi (Pilocarpus microphyllus stapf). In Vitro Cell. Dev. Biol.: Plant.

[7] Sharma L., Desai A., Sharma A. (2006). A thin layer chromatography laboratory experiment of medical importance. Biochem. Mol. Biol. Educ.

[8] Reddy B.S., Rozati R., Reddy B.V.R., Raman N.V.V.S.S. (2006). Association of phthalate esters with endometriosis in Indian women. BJOG.

[9] Mulija M., Indrayanto G., Cazes J. (2001). Steroid Analysis by TLC. Encyclopedia of Chromatography.

[10] Scott R.P.W. (2007). Thin Layer Chromatography.

[11] Nyiredy Sz., Meier B., Erdelmeier C.A.J., Sticher O. (1985). PRISMA: A Geometrical Design for Solvent Optimization in HPLC. J. High Resolut. Chromatogr. Chromatogr. Commun.

[12] Zhang Y.P., Zhang Y.J., Gong W.J., Gopalan A.I., Lee K.-P. (2005). Rapid separation of Sudan dyes by reverse-phase high performance liquid chromatography through statistically designed experiments. J. Chromatogr. A.

[13] Cimpoiu C., Jäntschi L., Hodişan T. (1998). A New Method for Mobile Phase Optimization in High-Performance Thin-Layer Chromatography (HPTLC). J. Planar Chromatogr. - Mod. TLC.

[14] Cimpoiu C., Hodisan T. (1999). Application of numerical taxonomy techniques to the choice of optimum mobile phase in high-performance thin-layer chromatography (HPTLC). J. Pharm. Biomed. Anal.

[15] Vasiljević T., Onjia A., Čokeša Đ., Laušević M. (2004). Optimization of artificial neural network for retention modeling in high-performance liquid chromatography. Talanta.

[16] Tran A.T.K., Hyne R.V., Pablo F. (2007). Optimisation of the separation of herbicides by linear gradient high performance liquid chromatography utilising artificial neural networks. Talanta.

[17] Cimpoiu C., Jäntschi L., Hodişan T. (1999). A New Mathematical Model for the Optimization of the Mobile Phase Composition in HPTLC and the Comparision with Other Models. J. Liq. Chromatogr. Relat. Technol.

[18] Jäntschi L., Hodişan S., Cimpoiu C., Ceteraş I. (2005). Analysis of Some Steroids by TLC Using Optimum Mobile Phases. Acta Univ. Cibin. - Ser. F Chem.

[19] Jäntschi L., Hodişan S., Cimpoiu C., Hosu A., Darvasi E., Hodişan T. (2007). Modeling of thin-layer chromatographic separation of androstane isomers. J. Planar Chromatogr. - Mod. TLC.

[20] Hodişan S., Cimpoiu C., Casoni D. (2004). Separation of Androstan Isomers by High Performance Thin Layer Chromatography (HPTLC) Using an Optimum Mobile Phase. Acta Univ. Cibin. - Ser. F Chem.

[21] Stoenoiu C.E., Bolboacă S. D., Jäntschi L. (2006). Mobile Phase Optimization Method for Steroids Separation. Appl. Med. Inform.

[22] Ettre L.S. (1993). Nomenclature for Chromatography. (IUPAC Recommendations 1993). Pure Appl. Chem.

[23] Bolboacă S.D., Jäntschi L. (2006). Pearson versus Spearman, Kendall's Tau Correlation Analysis on Structure-Activity Relationships of Biologic Active Compounds. Leonardo J. Sci.

[24] Jäntschi L., Bolboacă S.D. (2007). Triazines herbicidal assessed activity. Stud. Cercet. Stiint. - Ser. Biol., Univ. Bacau.

[25] Bolboacă S.D., Jäntschi L. (2008). Modelling the Property of Compounds from Structure: Statistical Methods for Models Validation. Environ. Chem. Lett.

